# Fatty Acid Contents and Stability of Oyster Nut Oil (*Telfairia pedata*) Compared to Flaxseed and Sunflower Oil

**DOI:** 10.1155/2021/9985910

**Published:** 2021-11-11

**Authors:** Emmanuel Mwakasege, Anna Treydte, Otmar Hoeglinger, Neema Kassim, Edna Makule

**Affiliations:** ^1^Department of Food Biotechnology and Nutritional Sciences. School of Life Science and Bio-Engineering, The Nelson Mandela African Institution of Science and Technology (NM-AIST), P.O. Box 447, Arusha, Tanzania; ^2^Department of Food Science and Technology. Mwalimu Julius K. Nyerere University of Agriculture & Technology, P.O. Box, 976 Musoma, Tanzania; ^3^Department of Sustainable Agriculture, Biodiversity Conservation and Ecosystems Management, The Nelson Mandela African Institution of Science and Technology (NM-AIST), P.O. Box 447, Arusha, Tanzania; ^4^University of Applied Sciences Upper Austria, 4600 Wels, Austria

## Abstract

The selection of healthy fats for consumption is important. Linoleic acid (LA) (omega-6) and alpha-linolenic acid (ALA) (omega-3) are essential polyunsaturated fatty acids required for the maintenance of good health; however, LA derivatives such as arachidonic acid (AA) are associated with the onset of inflammatory diseases, and both are prone to oxidation and deterioration. This study compared the fatty acid contents, peroxide value (PV), p-anisidine value (p-AV), and free fatty acids (FFA) of the oyster nut oil with refined sunflower, nonrefined sunflower, and flaxseed oil stored at 27°C for 40 days. Flaxseed oil had significantly high ALA content (59.8%) compared to 0.1-0.5% for oyster nut and sunflower oil brands. The LA content was high in sunflower brands (50.3-52.8%) compared to the oyster nut (48%) and flaxseed oil 14.7%. Oleic acid was lower in oyster nut oil (8.6%) and flaxseed oil 15.8% compared to sunflower brands (35.7-38.2%). As a consequence, oyster nut and flaxseed recorded higher PV of 4.35-2.88 mEq O_2_/kg and FFA 0.26-0.47% compared to sunflower brands. The p-AV recorded small values which were not significantly different in all samples. Although oyster nut is widely consumed by pregnant and lactating women across Africa, its keeping quality in nonrefined form is low compared to flaxseed and sunflower oil as shown in this study. Hence, the fatty acid contents in oyster nuts should be consumed in other alternative forms such as flour and roasted kernels rather than its oil when in nonrefined form. This study will enable the consumption balance of omega-6/omega-3 fatty acids and the keeping quality of oils which is key to health.

## 1. Introduction

Since the industrial revolution in the 1950s, fat consumption patterns have shifted towards commercial vegetable oils, nuts, and seeds high in saturated fatty acids and omega-6 polyunsaturated fatty acids [1]. The omega-6/omega-3 fatty acid consumption ratio has shifted from 1 : 1 to 20 : 1 causing the increase of cardiovascular diseases, obesity, diabetes, and other inflammatory diseases [2, 3]. Both omega-6 and omega-3 fatty acids are essential to our body, but omega-6 derivatives such as eicosanoids (AA) metabolites cause the onset of inflammation while omega-3 derivatives such as docosahexaenoic acid (DHA) and eicosapentaenoic acid (EPA) show inflammation suppression effects [3–5]. Balancing the consumption ratio of omega-6/omega-3 to 1 : 1 is said to be key for the maintenance of health. The World Health Organization (WHO) recommends an upper limit of only 9% of dietary energy to come from linoleic acid (LA) consumption which is 25 grams per day for men and 20 grams for women [5]. Hence, there is a need to select sources of fat with a balance of omega-6/omega-3 for consumption.

Furthermore, oil quality and stability are also affected by variations in fatty acid compositions and antioxidant contents [6, 7]. Despite their nutritional benefits, LA and ALA are more prone to oxidative deterioration during storage and high heat application [8]. The double bond structure in LA and ALA (PUFAs) is unstable [9]. It readily reacts with singlet oxygen forming oxidative by-products such as free radicals, hydroperoxides, and aldehydes causing detrimental health effects [10]. Therefore, for long-time storage and high heat applications, oilseeds with high monounsaturated fatty acids (MUFA) contents are preferred by the oil industry over oilseeds with high PUFA contents. Various methods are used to determine oil stability during storage or application [11]. These methods are used to evaluate the extent of oil deterioration by measuring indicative parameters. Factors such as percentage of free fatty acids (FFA), peroxide value (PV), amounts of *β*-carotene, type of fatty acids, and specific extinction coefficient are widely used. Others are changes in the number of phenols, tocopherols, and oxidative stability [7]. In most cases, these parameters are determined simultaneously to enable precision in the evaluation of oil stability.

Therefore, the selection of fats for consumption should be based on fatty acid contents and their stability during storage. Oyster nut (*Telfairia pedata*) is the oilseed plant of the family *Cucurbitaceae* cultivated and used as the source of oil in East and Central Africa [12, 13]. For the local tribes in Africa, oyster nuts have been regarded as traditional heritage food of high quality given to pregnant and lactating women to improve milk secretion or to enhance fast healing after childbirth [14, 15]. Despite being widely consumed in Africa and other parts of the world, there are no fatty acid contents or oil quality documentation to support its nutritional claims. In addition, sunflower (*Helianthus annuus*) and flaxseed (*Linum usitatissimum* L.) are common sources of commercial oil in the world [16–18]. Both sunflower and flaxseed contain high LA and ALA which can affect their keeping quality [8, 11]. Therefore, this study characterizes and compares fatty acid contents and oil stability of nonrefined oyster nut oil, nonrefined flaxseed oil, nonrefined sunflower oil, and refined sunflower oil. In most cases, oyster nut oil is locally pressed and utilized in nonrefined forms; hence, this study will compare the keeping quality of oils in their nonrefined forms. We hypothesized that oyster nut oil as the new source of oil contains a healthy fatty acid profile with more keeping quality compared to flaxseed and sunflower oil during storage in their nonrefined forms. This study will provide information on the effects of fatty acid contents on the stability of nonrefined oilseeds and enhance the informed selection of healthy fats for consumption.

## 2. Materials and Methods

### 2.1. Preparation of Oil Samples

Oyster nut (*Telfairia pedata*) kernels were dried at 60°C for 7 hours until the constant weight of the grated kernels was achieved before the extraction of oil. The oil was extracted using a laboratory oil press (Scheler brand) and stored. The other oils native flaxseed oil (*Linum usitatissimum* L.), refined sunflower oil (*Helianthus annuus*), and nonrefined sunflower oil were bought from the local market in Wels, Austria. All samples were relatively fresh with less than 1 week since extraction. The four oil samples were stored in a 1000 mL transparent capped bottle with approximately 10 cm^2^ of the atmosphere at the top. The samples were stored at 27°C for 40 days and periodically shaken before samples were withdrawn for further analysis. Fatty acid contents were analyzed immediately and after 40 days of storage, while p-anisidine value (p-AV), peroxide value (PV), and free fatty acid (FFA) contents were analyzed after every 7 days.

### 2.2. Fatty Acid Analyses

Derivatization of fatty acid methyl esters was done according to AOAC methods [19] with modifications. The first 10 mg dry oil was methylated with a mixture of 5 mL methanol/acetyl chloride at a ratio of 50 : 1 at 60°C for 4 hours. Afterward, 2.5 mL sodium hydroxide (60 g/L) was slowly added to stop the reaction. Methyl esters were extracted by shaking with 2 mL hexane, and 1000 *μ*L of clear upper phase was transferred in a GC vial for injection.

FAME was analyzed using thermo-trace gas chromatography connected with an autosampler (AS 2000) and flame ionization detector (FID) (Agilent Tech Inc., Wilmington, DE, USA). Injection volume was 2 *μ*L at 240°C. Helium was a carrier gas with a flow of 30 mL/min at a constant pressure of 120 kPa. The analytical separation was done by the Agilent capillary column (0.25 mm ID, DB23 60 m, and film thickness of 0.25 *μ*m). The temperature gradient for the oven was 0-3 min to 130°C, 6.5°C/min to 170°C, and 2.8°C/min to 215°C settled for 10 minutes. Then, 3°C/min to 240°C for 15 minutes. The FID airflow was 450 mL/min, at 280°C, hydrogen flow at 45 mL/min, and nitrogen held at 40 mL/min. Calibration was done using the external standard method, and data were analyzed using Chrom card Data system 2.8 from Thermo Finnigan. All reagents were of analytical grade, and analyses were done in duplicates.

### 2.3. Determination of Oil Stability

#### 2.3.1. Peroxide Value (PV)

Peroxide value was analyzed as described by Casal et al. [6] where 5 g of samples was measured in an Erlenmeyer flask and dissolved the sample by adding 25 mL of the 3 volumes of glacial acetic acid and 2 volumes of chloroform solvent mixture. Then, 0.5 mL of the saturated potassium iodide solution was pipetted to the sample and swirled on a magnetic mixer for precisely 60 seconds. Then, the solution was diluted with 25 mL of distilled water to stop the reaction. Then, 1 mL of 1% starch solution was added, followed by titration using the 0.01 sodium thiosulfate solution in the burette until the blue color disappeared.

(1) $$\begin{array}{c} \text{PN}=\frac{\left(a-b\right)*c*1000}{E}, \end{array}$$

where *a* is the total consumption of sodium thiosulphate standard solution, *b* is the consumption of sodium thiosulphate in a blind test, *c* is the concentration of used sodium thiosulphate solution, and *E* is the lipid weight.

#### 2.3.2. The Free Fatty Acids (FFA)

FFA was analyzed according to Frega et al. [20], where 15 g of the oil sample was weighed into an Erlenmeyer flask and dissolved in 50 mL (1 volume ethanol + 1 volume toluene) solvent mixture. A few drops of the phenolphthalein indicator solution were added, then titrated with the potassium hydroxide standard solution until the titration endpoint (permanent red coloring).

(2) $$\begin{array}{c} \text{AN}=\frac{a*c*56.1}{E}, \end{array}$$

where *a* is the consumption (mL) of potassium hydroxide solution, *c* is the concentration of the potassium hydroxide solution (mol/L), and *E* is the lipid weight (56.1 = molar mass (g/mol) potassium hydroxide).

(3) $$\begin{array}{c} \text{FFA}\%=\text{AN}*\frac{M\left(\text{FS}\right)*100}{56.1*1000}, \end{array}$$

where *M* (FS) is the molar mass of free fatty acids = 282 g/mol.

#### 2.3.3. p-Anisidine Value (p-AV)

The p-anisidine value was used to determine the secondary oxidation products (aldehydes) as described by Nielsen [21]. Photoelectric measurements of the increase of absorbance of grams of oil in isooctane were measured in a 10 mm cuvette at the wavelength of 350 nm. The sample was incubated for 8 minutes at 22°C and was measured before and after reaction with the p-anisidine reagent in the dark.

(4) $$\begin{array}{c} \text{AN}=\frac{100QV\left(1,2\left(E1-E2-E0\right)\right.}{M}, \end{array}$$

where *V* is the volume of dissolved sample, *M* is the weighed sample, *Q* is the amount of sample of measured solution (g/mol), *E*0 is the extinction of solution prior to reaction, *E*1 is the after reaction, and *E*2 is the blank solution.

### 2.4. Statistical Analyses

Analysis of variance (ANOVA) was used to compare oyster nut oil's fatty acid contents, PV, p-AV, and FFA to flaxseed oil, refined, and unrefined sunflower oils [8]. Pearson's correlation was used to establish the effects of storage time on the peroxide value (PV), p-anisidine value, and free fatty acid (FFA) contents in the four oil samples [6]. Post hoc tests using Tukey's multiple comparison tests were done to compare means. The criterion for significance was set at *p* < 0.05. All analyses were run using R-software version 3.3.

## 3. Results and Discussion

### 3.1. Fatty Acid Contents in Oyster Nuts, Flaxseed, Nonrefined Sunflower, and Refined Sunflower Oil

**Figure 1:**
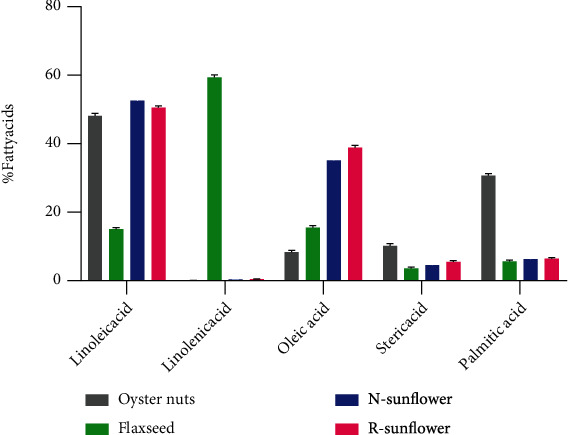
Bar graphs showing variations in average contents of the major fatty acid contents (%) in oyster nut oil, flaxseed oil, refined sunflower oil, and nonrefined sunflower oil.

The three types of oilseeds showed significant variations in fatty acid contents. As shown in Figure 1, the major fatty acids found in the four oil types were linoleic acid (C18:2 LA), linolenic acid (C18:3 ALA), oleic acid (C18:1), stearic acid (C18:0), and palmitic acid (C16:0). Among the three oil types, flaxseed oil had significantly (*p* < 0.05) lower linoleic acid (LA) contents (14.7%) compared to sunflower and oyster nut oil which contained 48-53%. Similar fatty acid profiles in flaxseed and sunflower oil were previously reported by Zhang et al., Bozan and Temelli, and Hasiewicz-Derkacz et al. [8, 10, 16]. Since the purpose is to reduce dietary energy from LA consumption to recommended values below 9% [5], in that regard, flaxseed oil displayed a healthy LA content compared to sunflower and oyster nut oil. LA is an essential fatty acid and the major fatty acid content in nuts, oilseeds, and most vegetable oils. Due to its abundance, in most cases, overconsumption occurs which affects the LA/ALA ratio. Hence, oil sources with low LA contents are more preferred for consumption. This has led the vegetable oil industry to focus breeding efforts towards sunflower varieties with less LA and more oleic acid [22].

Unlike LA contents, ALA contents were significantly (*p* < 0.05) higher in flaxseed oil (59.5%) compared to (0-0.5%) in oyster nuts and sunflower oils. In some oyster nut oil samples, ALA was undetected by GC. This indicates that oyster nut is not a good source of ALA and should always be consumed along with other ALA sources such as leafy green vegetables and fish [1]. Although oyster nut is regarded as high-nutrient oilseed and used as a natural medicine in Africa, its consumption should be limited due to high LA contents. Furthermore, flaxseed oil is a good source of ALA even though the ability of the body to convert ALA into its derivatives such as ADH and EPA is limited [2]. This results in the unavailability of ADH and EPA regardless of high flaxseed consumption compared to consumption of fish [4].

Oleic acid is a major monounsaturated fatty (MUFA) acid in oilseeds. Apart from contributing to (high-density lipoprotein) HDL contents in blood, oleic acid is important for the stability of oils. The single double bond structure makes oleic acid less prone to oxidation enhancing the shelf life of the oil [6]. In this study, oleic acid contents were significantly (*p* < 0.001) higher in the two sunflower brands compared to flaxseed and oyster nut oil. In this regard, sunflower oil is regarded as the best oil choice with a healthy fatty acid profile compared to flaxseed and oyster nut oil.

Palmitic acid (C16:0) which constitutes the saturated fatty acids (SFAs) was significantly (*p* < 0.05) high in oyster nut oil (31.2%) compared to the range of 5.8 to 6.5 in flaxseed and sunflower oils. Furthermore, steric acid (C18:0) which also constitutes SFAs was slightly high (10.8%) in oyster nut compared to flaxseed and sunflower oil (3.5-5.2%). High consumption of SFAs is associated with a rise in low-density lipoprotein in the blood which results in cardiovascular diseases (CVD) [23, 24]. This result suggests that although oyster nuts contain high polyunsaturated fatty acids, its consumption should be supplemented with other sources of ALA such as fish, flaxseed, chia seeds, or olive oil. This will enhance the consumption balance for omega-6 and omega-3 fatty acids.

### 3.2. Changes in Fatty Acid after 40-Day Storage Period

Despite significant changes in PV and FFA contents, there were only slight changes in fatty acid contents after 40 days. As shown in Table 1, there was a slight increase in oleic acid and a decrease in polyunsaturated fatty acids (linoleic and linolenic acid) in oyster nuts and flaxseed oil. These compositional changes are attributed to hydrolysis and oxidation of triglyceride double bonds to form primary and secondary oxidation products such as peroxides and aldehydes [25]. Oleic acid (MUFA) is relatively less prone to deterioration compared to PUFAs [9]; hence, a percentage increment in oleic acid was expected. The same results were reported by Casal et al. [6] where a slight percentage increment in oleic acid contents was observed at the initial stages of heating olive oil at 170°C. Crapiste et al. [26] reported a reduction in PUFA contents in sunflower oil blends during accelerated storage at 67°C. There were no significant changes (*p* < 0.05) in SFA contents during the storage period. This indicated stability SFAs during storage at room temperature. Generally, both types of sunflower oil exhibited more stability compared to oyster nut and flaxseed oil. This stability might be associated with high antioxidants and less PUFA contents in sunflower oil as compared to flaxseed and oyster nut oil reported by Zhang et al. [8]. Oyster nuts and flaxseed oil exhibited similar storage and stability characteristics, which is reflected in their high PUFA and less MUFA contents. This result indicates the stability of the three oil samples during 40 days of storage even though the hydrolysis of triglycerides especially in highly unsaturated oils indicates the nutritional reduction trend when stored for longer periods.

### 3.3. Changes in Free Fatty Acid (FFA) Contents

**Figure 2:**
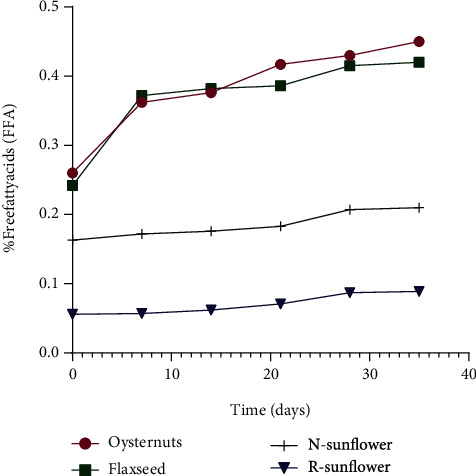
Line graphs showing variations in free fatty acid (FFA) of oyster nut oil, flaxseed oil, refined sunflower oil, and nonrefined sunflower oil samples stored at 25°C for a 40-day period.

Due to high temperature, enzymatic activities, or high moisture contents in oil, hydrolysis of triglycerides occurs due to the detachment of fatty acid tails from glycerol molecules resulting in the formation of FFAs [7]. FFAs are highly susceptible to oxidation; hence, they indicate deterioration in oils [6]. During the 40-day storage period, all four oil samples showed no significant difference (*p* < 0.05) in the amount of produced FFAs as shown in Figure 2. Still, the trends indicated high FFA values in unrefined oyster nut oil (0.26 to 0.39%) and flaxseed oil (0.42 to 0.47%) compared to those (0.16 to 0.2% and 0.05 to 0.08%) in unrefined and refined sunflower oil, respectively. The FFA contents are highly reduced by saponification during the refining process, so FFA contents were in low amounts (0.05%) in refined sunflower oil compared to nonrefined oyster nut, flaxseed, and sunflower oil. In addition, refined oils contain fewer enzymes and metals which can influence the hydrolysis of triglycerides. Although oyster nut oil was freshly prepared through a cold press, its initial FFA contents were higher and similar to that of flaxseed oil; this reflects on the benefits of oil refining which reduces FFAs and other impurities enhancing the stability of oil [27].

These results reflect on the importance of refining the oils before storage since refined sunflower oil showed significant stability compared to unrefined sunflower oil. Both flaxseed and oyster nut oils showed less stability compared to nonrefined sunflower oil. In the local context, oyster nuts are pressed and stored in nonrefined forms which reflects their instability. Therefore, the keeping quality can be improved by though cleaning during preparation and proper drying to reduce moisture before the kernels are pressed to oil in the local context.

### 3.4. Changes in Peroxide Value (PV)

**Figure 3:**
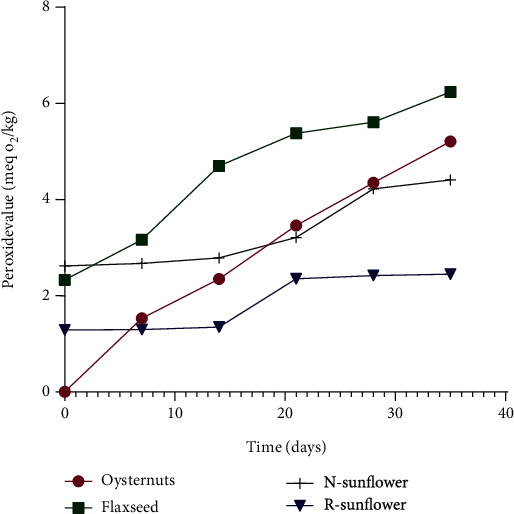
Line graphs showing variations in peroxide values (PV) of oyster nut oil, flaxseed oil, nonrefined sunflower oil, and refined sunflower oil samples stored at 25°C for a 40-day period.

Estimation of primary and secondary oxidation products was done using peroxide value (PV) and p-anisidine value, respectively. Peroxide value (PV) is a popular oil oxidation indicator that reflects on the formed primary oxidation products such as hydroperoxide compounds [8]. In this study, the PV for oyster nut oil was not significantly different (*p* < 0.05) from that for flaxseed oil as shown in Figure 3. Existing similarities in fatty acid profiles might cause this. Both oil samples were nonrefined and more prone to oxidation. The peroxide values (PV) increased by 4.35 mEq O_2_/kg in the oyster nut oil sample, while flaxseed, nonrefined, and refined sunflower oils recorded 2.88 mEq O_2_/kg, 1.61 mEq O_2_/kg, and 1.13 mEq O_2_/kg increase, respectively. According to European Union standards, virgin oils should have PV values below 20 mEq O_2_/kg, 10 mEq O_2_/kg for vegetable oils, and 15 mEq O_2_/kg for refined oils [6]. During the 40 days, all oil samples were within recommended specifications. Additionally, both oil samples contain high polyunsaturated fatty acids (PUFA), which are more prone to oxidation. According to our results, oyster nut oil contains 47% linoleic acid (PUFA) and only 8% oleic acid (MUFA), while flaxseed oil contains 73% PUFA and only 16% oleic acid (MUFA). Sunflower oil had a higher (38%) MUFA content which is associated with oil stability. On antioxidant contents, sunflower oil has been reported to contain higher 41.08 mg/100 g vitamin E compared to 2.03 mg/100 g vitamin E, 23.34 mg/100 g vitamin A, and 2.68 mg/100 g beta-carotenes reported in oyster nut oil by Musalima et al. [28]. These results reflect on the implications of high PUFA contents in oyster nut oil stability and highlight the need for refining and antioxidant supplementation during long-time storage.

### 3.5. Changes in p-Anisidine Value (p-AV)

**Figure 4:**
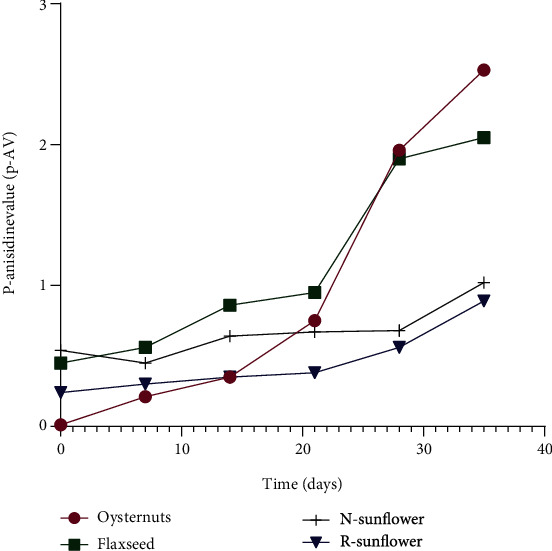
Line graphs showing variations in p-anisidine values (p-AV) of oyster nut oil, flaxseed oil, nonrefined sunflower oil, and refined sunflower oil samples stored at 27°C for 40 days.

As expected, the photometric measurement of anisidine values at 350 nm wavelengths was relatively low for all oil samples. The anisidine value gives an estimation of secondary oxidation products such as 2-alkanals, 2,4-dienals, and unsaturated aldehydes [29]. Since the experiment was conducted for 40 days using relatively fresh oil samples, the anisidine values were low and the same in initial periods for all oil samples ranging from 0 to 1.0 mEq O_2_/kg. As shown in Figure 4, the p-AV values started to get higher in flaxseed and oyster nut oils after 30 days, which indicates high secondary oxidation product formation in later stages due to high peroxide contents.

Generally, p-AV values were lower in all samples during the first 20 days of storage and were similar to values reported in *Lannea kerstingii* oil 0.8 and *Salvia hispanica* L which was 0.3 total p-AV [30]. While p-AV values remained constant for sunflower samples after 20 days of storage, in flaxseed and oyster nut oils, the secondary oxidations were elevated. The values were elevated to 3.5 for oyster nut and 2.0 for flaxseed oil after 40 days. The sudden increase in p-AV values might be due to the instability of double bonds in flaxseed and oyster nut oils compared to sunflower oil. In addition, the high p-AV is attributed to the excess amount of FFA after 20 days which is more prone to secondary oxidation. These results indicate the importance of performing both PV and AV stability tests simultaneously since the p-AV indicates the true measure of formed hydroperoxides which are converted to secondary aldehyde products.

## 4. Conclusion

Oyster nut seeds and oil are regarded as nutritious foods and consumed by pregnant and lactating mothers across Africa, and in most cases, the oil is locally pressed and stored in nonrefined forms. The oyster nuts contain a nutritious and quality fatty acid profile that is high in PUFA contents including a high amount of omega-6 fatty acids. During the 40-day storage at 27°C, the fatty acid profile of oyster nuts showed no significant difference. In addition, the study shows the instability of the oyster nut oil as expected due to high PUFA contents forming high free fatty acids, peroxide value, and p-anisidine value compared to control samples during the 40-day storage. Due to the instability of oyster nut oil during storage, the nuts can be consumed in alternative forms such as flour or roster whole nuts to benefit from their healthy fatty acid profile.

## Figures and Tables

**Table 1:** Average (±SD) of the major fatty acid contents (%) of oyster nuts oil, flaxseed oil, nonrefined sunflower oil, and refined sunflower oil measured at day 1 and after 40 days of storage at 27°C.

Fatty acids	Day 1				Day 40			
	Oysternut	Flaxseed	N-sunflower	R-sunflower	Oysternut	Flaxseed	N-sunflower	R-sunflower
Linoleic acid	48.8 ± 1.1^C^	14.1 ± 0.5^C^	52.7 ± 0.2	50.3 ± 0.3	47.5 ± 1.3^C^	13.7 ± 0.4^C^	52.5 ± 0.2	50.0 ± 0.2
Linolenic acid	0.1 ± 0.0	59.8 ± 0.5^C^	0.3 ± 0.0	0.5 ± 0.0	0.1 ± 0.1	57.9 ± 0.4^C^	0.3 ± 0.1	0.4 ± 0.0
Palmitic acid	31.7 ± 1.0	5.8 ± 0.5	6.3 ± 0.3	6.5 ± 0.9	32.5 ± 1.3	5.6 ± 0.4	6.3 ± 0.2	6.2 ± 0.7
Stearic acid	10.8 ± 1.0	3.5 ± 0.6	4.7 ± 0.6	5.2 ± 0.3	10.9 ± 0.3	3.6 ± 0.5	4.9 ± 0.4	5.3 ± 0.4
Oleic acid	8.6 ± 0.6^C^	15.8 ± 0.7^C^	35.7 ± 0.1	38.2 ± 0.5	9.7 ± 1.2^C^	16.7 ± 0.3^C^	35.5 ± 1.2	38.5 ± 0.9
SFAs	43.1 ± 0.2	9.3 ± 0.6	11.0 ± 0.3	11.7 ± 0.6	43.4 ± 0.6	9.2 ± 0.7	11.2 ± 0.5	11.6 ± 0.9
MUFAs	8.6 ± 0.1^C^	15.8 ± 0.7^C^	35.7 ± 0.1	38.2 ± 0.5	9.7 ± 0.2^C^	16.7 ± 0.3^C^	35.5 ± 0.6	38.5 ± 0.8
PUFAs	48.9 ± 0.4^C^	73.9 ± 0.3^C^	53.0 ± 0.2^C^	50.7 ± 0.3	47.6 ± 0.4^C^	71.6 ± 0.6^C^	52.8 ± 0.2*C*	50.4 ± 0.3

Values with the letter “C” in superscript indicate significant changes (*p* < 0.05) of the same sample after 40 days of storage.

## Data Availability

Data is available in the manuscript and from the authors.
